# Can the Edinburgh Postnatal Depression Scale-3A be used to screen for anxiety?

**DOI:** 10.1186/s40359-021-00623-5

**Published:** 2021-08-07

**Authors:** Johanne Smith-Nielsen, Ida Egmose, Katrine Isabella Wendelboe, Pernille Steinmejer, Theis Lange, Mette Skovgaard Vaever

**Affiliations:** 1 Department of Psychology, University of Copenhagen, Copenhagen, Denmark; 2 Department of Public Health, University of Copenhagen, Copenhagen, Denmark

**Keywords:** Postnatal, Perinatal, Depression, Anxiety, Screening, EPDS-3A

## Abstract

**Background:**

Anxiety in the ante- and postnatal period is prevalent, often co-occurs with depression, and can have adverse consequences for the infant. Therefore, perinatal mental health screening programs should not only focus on depression but also on detecting anxiety. However, in many already implemented perinatal screening programs, adding extra screening instruments is not feasible. We examine the utility of a subscale of the Edinburgh Postnatal Depression Scale (EPDS) consisting of items 3, 4, and 5 (EPDS-3A) for detecting anxiety in new mothers.

**Methods:**

We used confirmatory factor analysis (CFA) to confirm the presence of the EPDS-3A found in a previous study (*n* = 320) where exploratory factor analysis (EFA) was used. For the CFA we used a sample of new mothers (*n* = 442) with children aged 2–11 months recruited from the same population from which mothers for the previous study was recruited. Three models were tested and compared. Receiver operating characteristics of the EPDS-3A were investigated in relation to anxiety caseness status on the combined sample (*N* = 762). Sample weighing was used to match the dataset to the target population. Cross tabulation was used to investigate the proportion of anxiety cases identified by the EPDS-3A above those identified with the total EPDS.

**Results:**

The presence of the EPDS-3A was confirmed. An EFA-driven, two-dimensional 7-item model showed the best data fit with one factor representing the anxiety subscale consisting of items 3, 4, and 5. An EPDS-3A score of ≥ 5 was the most optimal for identifying cases of anxiety (sensitivity: 70.9; specificity: 92.2; AUC: 0.926). Further, we found that the EPDS-3A identifies an additional 2.5% of anxiety cases that would not have been identified with the total EPDS.

**Conclusions:**

The EPDS-3A can be used as a time-efficient screening for possible anxiety in ante- and postnatal mothers. However, adding the EPDS-3A to routine screening with the total EPDS does not lead to a substantial increase in the number of women identified. In line with previous studies, this study confirms that the EPDS identifies anxiety in addition to depression. Therefore, assessment and treatment adjusted to the specific emotional difficulties is imperative.

**Supplementary Information:**

The online version contains supplementary material available at 10.1186/s40359-021-00623-5.

## Background

It is generally accepted that the detection of ante- and postnatal depression is crucial for the prevention of adverse effects on maternal and infant mental health [1]. Accordingly, recommendations for screening programs in primary care often include screening for antenatal and postnatal depression [2]. However, researchers have begun to question whether screening for mental health problems in the perinatal period should be limited to detection of depression [3, 4].

Anxiety is prevalent in the perinatal period [5, 6] and can have significant impacts on the mother as well as on the offspring [7–9]. Moreover, perinatal anxiety often co-occurs with depression [3]. For example, a population-based study of postnatal women (*N* = 522) found that 52% of those, who reported depressive symptoms, also presented with high levels of anxiety [10], another study (*N* = 4451) found a prevalence of postnatal anxiety of 18% out of which 35% also reported symptoms of depression [5], and a recent meta-analysis shows that the prevalence of co-morbid depression and anxiety disorder is 8.2% during the first 24 weeks postnatal [3]. Based on such results, it may be argued that perinatal screening programs should include the detection of women with high levels of anxiety [3, 11].

Yet, when implementing public health screening programs, the screening program must be time and resource-effective, and in most primary healthcare settings, it is not feasible to add extra instruments to already implemented screening programs. Taking a pragmatic approach, in the current study, we investigate the utility of using a subscale of the Edinburgh Postnatal Depression Scale (EPDS: [12]) to screen for anxiety in postpartum mothers.

The EPDS is one of the most validated perinatal screening instruments [13] and is implemented in many primary healthcare settings around the world, including the Danish where the current study was conducted. Although the EPDS was intended to be unidimensional, studies have demonstrated that the scale is multidimensional with at least two factors, including an ‘anxiety factor’ as well as a depression factor. Most studies find that the anxiety factor includes items 3, 4, and 5 (for a review, see Kozinszky et al. [14]). This factor has been referred to as the EPDS-3A [1]; henceforth, this term is used in the current paper. In a validation study of the Danish version of the EPDS [15], using exploratory factor analysis (EFA), we also found the EPDS to be multidimensional with a sub-factor consisting of items 3, 4, and 5.

Some authors suggest that the EPDS-3A can be used to screen for anxiety: Matthey (2008) [16] used principal components analysis to confirm the presence of items 3, 4, and 5 as a sub-factor and examined Receiver Operating Characteristics (ROCs) in relation to fulfilling diagnostic criteria for an anxiety disorder (*N* = 238). He found a cutoff score of ≥ 6 to be effective in detecting at least one anxiety disorder (sensitivity: 66.7%; specificity: 88.2%). Eighteen women met criteria for a diagnosis of anxiety, and 11 (61%) of these were not detected using the total EPDS cutoff score. Another study [17] also used principal components analysis to confirm the EPDS-3A and correlated EPDS-3A scores with scores on a psychosocial risk factors questionnaire. A score of ≥ 4 was suggested as the optimal cutoff as it identified the top-quartile of the sample. In this study, the proportion of women identified with the EPDS-3A, above those identified with the total EPDS, was not investigated. Stasik-O’Brien et al. [18]identified the EPDS-3A with EFA and used the cutoff suggested by Matthey [16] to detect mothers with emotional distress and anxiety symptoms in an at-risk sample. In this study, no measure of anxiety was used to confirm the positive EPDS-3A score. Of the 200 participants, 43 (21.9%) scored above EPDS-3A cutoff and ten (23.3%) of these did not screen positive on the total EPDS.

In contrast, two studies conclude that the EPDS-3A does not perform well enough to be used for screening for anxiety in the postnatal period. Among a range of screening tools, Fairbrother et al. [19] evaluated the EPDS-3A for the detection of postnatal anxiety (*N* = 360). A diagnostic interview was used as the criteria. To be recommended for widespread clinical application, the authors argued that a screening tool should evidence an area under the ROC curve (AUC) ≥ 0.8. The EPDS-3A performed slightly better than the total EDPS for detecting anxiety (AUCs: 0.757 vs. 0.744). However, with an AUC of 0.757, the authors conclude that the accuracy of the EPDS-3A was too low to recommend to be used to screen for anxiety. In a community sample (*N* = 550) [20] AUC for the EPDS-3A (which in this study consisted of items 3, 4, 5 along with item 10) was compared with AUC for the total EPDS for detecting clinical levels of anxiety measured using the Spielberger State-Trait Anxiety Inventory (STAI-6 [21]) as criteria. While AUC for the EPDS-3A was 0.729, it was 0.811 for the total EPDS, indicating that the total EPDS performed better than the EPDS-3A in terms of discriminating anxiety cases from non-cases. Similar results appeared when leaving out item 10 from the EPDS-3A. The authors conclude that adequate screening for anxiety requires an additional effort on top of the EPDS.

In sum, the evidence for the utility of using the EPDS-3A in routine screenings is mixed. The three studies advocating for using the EPDS-3A are limited by the use of different methodologies, relatively small sample sizes, and importantly, only one of them [16] validated the scale against anxiety casesness status.

Therefore, research is needed to inform decisions on whether the EPDS-3A could be used as a ‘good enough’ anxiety screener if including an extra instrument in a screening program is not feasible. Without adding extra time or burden on the screened woman, using the EPDS-3A could provide an efficient method for the detection of women in need of treatment who otherwise might be overlooked.

In the present study, we addressed the following research questions: (1) Can the Danish EPDS-3A be confirmed in a new sample of postnatal mothers recruited from the same population from which participants were recruited for the Dansih validation of the EPDS [15]?; and (2) can the EPDS-3A be used as an acceptable screening instrument in the detection of anxiety cases? We addressed this question by (a) evaluating ROCs of the EPDS-3A in relation to anxiety caseness status, and (b) investigating whether the EPDS-3A increases identification of women in need for further assessment and treatment above those identified by the total EPDS score.

## Methods

### Procedure and sampling

This study was part of a larger research project, the Copenhagen Infant Mental Health Project, that comprises the evaluation of several screening instruments for use in primary care, including a Danish validation of the EPDS published previously [15], and a treatment trial [22]. In the present study, we used two datasets: The dataset used for the Danish EPDS validation (data collected June 2015–July 2017: ‘original EPDS validation sample’) and a new dataset for which data was collected between August 2017 and June 2019 (‘new sample’).

Participants were recruited via public health visitors during routine home visits. At two months postpartum, and in some cases also at later visits, all mothers in Copenhagen are offered to be screened with the EPDS. To ensure recruitment of a sufficient number of women scoring in the high range of the EPDS, an oversampling strategy was used, similar to that used in other studies [23]. Accordingly, all mothers scoring 10 or more at the routine screening were invited to participate in the study. Additionally, to cover the whole range of the EPDS, a subgroup of health visitors invited women scoring 0–9 (for a detailed description of recruitment sampling strategy, see [15]). A psychologist conducted a second home visit where written informed consent was obtained, questionnaires on sociodemographic information were filled in, and the EPDS was administered again. The EPDS data collected during this visit was used in the present study. Participants received an online survey that included an anxiety measure. From August 2017, the protocol for the psychologist’s home visit was changed so that only mothers who were enrolled in the treatment trial received the online survey. Therefore, anxiety data were available for a smaller number of women than EPDS and sociodemographic data. Inclusion criteria were: mother at least 18 years old, speaks and reads Danish, and infant between 2 and 10 months.

### Measures

*Edinburgh Postnatal Depression Scale* [12] is a well-validated 10-item self-report questionnaire (range 0–30) designed to screen for possible depression in new mothers [See also Additional file 1: Edinburgh Postnatal Depression Scale]. In the Danish validation study of the EPDS [15], a cutoff score of ≥11 was found to be the most efficient to detect depression according to both ICD-10 and DSM-5 diagnostic criteria for depression (sensitivity: 79.2 and 78.2 respectively; specificity: 94.4; PPV: 49.0). This corresponds to findings from a recent meta-analysis of the accuracy of the diagnostic accuracy of the EPDS using individual participant data from 58 studies (*N* = 15,557) finding a cutoff of 11 or more to maximize combined sensitivity and specificity across reference standards [24]. Each item be can be scored from 0 to 3, and thus, the range of the EPDS-3A subscale is 0–9. In the current sample, Cronbach’s alpha for the full EPDS was 0.88.

*Hopkins Symptom Check-List, SCL-63* [25, 26] was used to establish anxiety caseness status. The questionnaire includes 63 items each rated on a five-point Likert Scale ranging from 0 (not at all) to 4 (extremely). The timeframe is the past week. The SCL-63 was validated in a Danish adult population (*N* = 1152) [25]. National norms and cutoffs have been established in a population sample of 2040 adult Danish women [26]. We used the cutoff for Danish women of the anxiety scale to define anxiety caseness. The cutoff indicates the demarcation between normal distress and clinical anxiety cases, which is in contrast to diagnostic systems, where classifications are based on diagnostic symptom criteria and not on quantitative measures of symptom severity with population norms. Cronbach’s alpha for the SCL anxiety subscale was 0.86 in this sample.

### Data analysis

#### Research question 1

Because there are several validated structures for the EPDS [14] and as EPDS-3A has already been identified with EFA in a Danish population of postnatal women [15], we used confirmatory factor analysis (CFA) to confirm the Danish EPDS-3A in the new sample [27]. IBM AMOS 26.0 [28] was used to conduct CFA as well as preliminary analyses to CFA.

#### Preliminary analyses

Prior to CFA, we used Mahalanobis Distance to screen for multivariate outliers. Multivariate normality kurtosis coefficient was assessed using a critical ratio of 5.0 as threshold as indicated by AMOS [28]. Since the presence of outliers and non-normal distribution of data can potentially cause inflated model fit statistics and standard error bias, we applied robust methods to handle both multivariate outliers and non-normality [29] as suggested by previous research [30]. Data screening indicated the presence of outliers and non-normal distribution of data (see also results) and therefore, we used bootstrapping as a robust solution for these violations [31]. The Bollen–Stine bootstrap procedure has been shown to perform just as well as robust maximum likelihood procedures and to perform effectively, despite excessive non-normality [29], and was therefore applied.

#### CFA

We compared a unidimensional model consisting of all 10 EPDS items with two different two-dimensional models to determine which measurement model best fitted the data. The tested models were: (1) a 10-item, one factor model, (2) a 7-item, two-factor model based on the EFA results from the Danish validation of the EPDS, and (3) an alternative two-factor model based on results from previous studies [32]. The initial EFA of the Danish EPDS suggested a three-factor structure where item 10 (the ‘self-harm item’) emerged as a separate third factor in addition to a depression factor consisting of items 1, 2, 8, and 9, and the anxiety factor consisting of items 3, 4, and 5. However, as also reported previously [15], parallel analysis did not firmly establish whether the correct number of factors were two or three, and in terms of proportion of explained variance, the two first factors were far more important than the third “one-item factor” that explained less than 10% of the variance. As factors with less than three items are weak and unstable [27], in the present study, in model 2 we omitted the third factor consisting of item 10. In Model 2, we also omitted items 6 and 7 because they cross-loaded on factor 1 and 2 in the EFA, thus resulting in a 7-item two-dimensional model. In the alternative 10-item two-factor model (Model 3, based on [﻿^32^]), we included all items in the depression factor except from items 3, 4, and 5.

The CFAs were conducted using maximum likelihood estimation with and without bootstrapping (set at 200 samples) and the Bollen–Stine bootstrap *p*-value to assess fit in addition to model fit indices [29]. These included: χ^2^ -test statistics (χ^2^/df), Comparative Fit Indices (CFI), Non-Normed Fit Index, or Tucker Lewis Index (TLI) and Root Mean Square Error of Approximation with accompanying 90% confidence interval (RMSEA). A good model fit is obtained when χ^2^/df ≤ 3, CFI and TLI > 0.90, and RMSEA < 0.08. With χ^2^/df values < 2 and RMSEA < 0.05, the model has an excellent fit. With χ^2^/df values below 2 and RMSEA < 0.05, the model has an excellent fit [33, 34]. Additionally, Modification Indices (MI) for chi-square change were considered for each model, and item errors were allowed to covary based on (a) MI values indicative of the greatest chi-square improvement, (b) theoretically meaningful covariances (such as similar or reversed items), and (c) if the items loaded on the same factor [28]. Factor loading threshold was established at 0.40 for moderate loadings [35]. Finally, for model comparison we evaluated the Akaike’s Information Criterion (AIC) and the Bayesian Information Criterion (BIC), with lower values indicative of a superior model [36]. Following model specification by CFA, internal consistency of the superior model was examined using Chronbach’s Alpha. However, as alpha for a scale comprising of more than one dimension has been demonstrated to underestimate reliability, in addition, construct reliability was examined using McDonald’s coefficient omega (ω) and hierarchical omega coefficient (h − ω) [37, 38]. This was calculated using an OMEGA macro extension for IBM SPSS 26 [39].

#### Research question 2

All subsequent analyses were conducted in R version 3.6.3 on a dataset combined of the original EPDS-validation sample and the new sample, and this data set was reweighted to match the target population. The calculation of sample weights was based on EPDS scores from 4931 postnatal women screened by health visitors in Copenhagen effectively giving us the population-wide distribution of EPDS scores (see also, [15]).

To define anxiety caseness status to be used as the criteria in the ROC analysis of the EPDS-3A, we used the raw score cutoff for the Danish SCL-63 anxiety scale (=1.15) [26]. Sensitivity, specificity, positive predictive value (PPV), negative predictive value (NPV), and AUC for all relevant cutoffs were computed directly from the reweighted data for anxiety caseness status. Confidence intervals were computed by embedding the calculations in a weighted logistic regression which provided confidence intervals corrected for weights. Because data-driven selections of optimal cutoffs may lead to overestimates of sensitivity and specificity [40], prior to analysis, we set criteria for what we considered acceptable levels of sensitivity and specificity when the EPDS-3A is used for first-phase screening purposes in addition to the full EPDS. Thus, to not overwhelm clinical services with many inappropriate referrals, we prioritized a high specificity (≥ 90%) while still aiming at a sensitivity that ensured the detection the majority of anxiety cases (≥ 70%).

Cross tabulation was used to investigate the proportion of anxiety cases identified by the EPDS-3A but not by the total EPDS.

**Table 1:** Sample characteristics

	Original EPDS validation sample (*n* = 320)	New sample (*n* = 442)	Combined sample (*N* = 762)
Maternal age, mean (*SD*)	32.2 (4.6)	31.9 (4.9)^a^	32.1 (4.8)^a^
Range	21–44	19–49	19–49
Infant age in weeks, mean (*SD*)	15.8 (8.2)	14.0 (7.3)	14.8 (7.7)
Infant age 6–14 weeks	209 (65.3)	313 (70.8)	522 (68.5)
Infant age 15–20 weeks	57 (17.8)	76 (17.2)	133 (17.5)
Infant age 21–30 weeks	29 (9.1)	32 (7.2)	61 (8.0)
Infant age 31–51 weeks	25 (7.8)	21 (4.8)	46 (6.0)
Infant sex, boys, *n* (%)	172 (53.8)	221 (50.0)	393 (51.6)
Infant gestational age, mean (*SD*)	39.7 (2.0)	39.5 (2.0)	39.6 (2.0)
Range	29.3–42.3	27.4–42.4	27.4–42.4
Infant prematurity, *n* (%)	19 (5.9)	38 (8.6)	57 (7.5)
Maternal country of origin			
Danish, *n* (%)	269 (84.1)	320 (72.4)	589 (77.3)
Immigrant, *n* (%)	31 (9.7)	37 (8.4)	68 (8.9)
Descendants of immigrants, *n* (%)	9 (2.8)	2 (0.5)	11 (1.4)
Missing, *n* (%)	11 (3.4)	83 (18.8)	94 (12.3)
Mother single parent or not			
Married or living with partner, *n* (%)	287 (89.7)	332 (75.1)	619 (81.3)
Single, *n* (%)	14 (4.4)	18 (4.1)	32 (4.2)
Other, *n* (%)	8 (2.5)	9 (2.0)	17 (2.2)
Missing, *n* (%)	11 (3.4)	83 (18.8)	94 (12.3)
Primiparous, *n* (%)	192 (60.0)	272 (61.5)	464 (60.9)
Missing	21 (6.6)	82 (18.6)	103 (13.5)
Maternal ISCED level of education			
Level 1–3, *n* (%)	29 (9.1)	26 (5.9)	55 (7.2)
Level 4 and 5, *n* (%)	37 (11.6)	30 (6.8)	67 (8.8)
Level 6, *n* (%)	111 (34.7)	141 (31.9)	252 (33.1)
Level 7 and 8, *n* (%)	133 (41.6)	159 (36.0)	292 (38.3)
Other or missing, *n* (%)	10 (3.1)	86 (19.5)	96 (12.6)

*ISCED* International Standard Classification of Education (UNESCO). Level 1–3 = Lower secondary or less. Level 4 = post secondary. Level 5 = short cycle tertiary. Level 6 = Bachelor’s degree or equivalent. Level 7 = Master’s degree. Level 8 = Doctoral degree or equivalent

^a^Age is missing for 24 mothers

## Results

In the new sample, EPDS data was available for 442 women. The combined dataset comprised 762 women (including the original EPDS-validation sample, *N* = 320). Sample characteristics are shown in Table 1. Anxiety data was available for 532 women. Of these, 161 (30.26%) presented with clinical levels of anxiety. In the combined dataset, mean total EPDS score was 11.25 (*SD* = 5.58; range = 0–28) and for the EPDS-3A, it was 4.56 (*SD* = 2.23; range: 0–9).

### Research question 1: confirmation of the EPDS-3A

#### Preliminary analyses

Data screening revealed 63 multivariate outliers and because reviewing data showed no outliers or data inaccuracy, outliers were not omitted from the data set. Kurtosis and skewness scores for each item fell between + 2 and − 2, except from item 10 (“The thought of harming myself has occurred to me”). Multivariate normality kurtosis coefficient was 8.751, with a critical ratio of 5.938, which was above the threshold of 5.0. In sum, data screening indicated the presence of outliers and non-normal distribution of data, potentially causing inflated model fit statistics and standard error bias. The Bollen–Stine yielded a p-value for estimation of overall model fit, with a threshold of *p* ≥ .05 indicative of a model with excellent data fit [29, 41].

#### CFA results

CFA results are presented in Table 2. Fit indices of Model 1, the unidimensional, 10 item model of the EPDS, initially revealed a poor model fit: χ^2^/df (157.907/35) = 4.521, *p* < .000, CFI = 0.916, TLI = 0.892, RMSEA = 0.089 (0.075 –0.104). Bollen–Stine *p* = .005, indicating poor overall model fit. Modification Indices (MI) indicated covariances between errors for item 1 (“I have been able to laugh and see the funny side of things”) and 2 (“I have looked forward with enjoyment to things”), item 4 (“I have been anxious or worried for no good reason”), and 5 (“I have felt scared or panicky for no good reason”), and between item 8 (“I have felt sad or miserable”) and 9 (“I have been so unhappy that I have been crying”). After inspection of the suggested MI and item properties, we added error covariances between these items. As a result, the model fit indices improved to a close to excellent fit, however, Bollen–Stine *p* = .005 suggested an overall poor fit. Factor loadings were significant and moderate to high (0.52 0.75), except for item 10 (self-harm), which had a factor loading of 0.35. CFA for model 2, the EFA-driven two-factor, 7 item model, demonstrated good fit indices for the CFI = 0.952 and TLI = 0.920, but not for the χ^2^/df statistics (59.679/13) = 4.592, *p* < .000, or RMSEA = 0.90 (0.68–0.114) or the Bollen–Stine *p* = .005. However, when adding suggested error covariances between item 1 and 2 and item 8 and 9 on the depression factor, the model had an excellent fit across all indices (Table 2). All item factor loadings were moderate to high (between 0.61 and 0.75) and significant onto their respective factor. Model 3, the alternative two-dimensional, 10 item model revealed an overall acceptable fit, except for χ^2^/df (118.422/34) = 3.483, *p* < .000, CFI = 0.942, TLI = 0.924, RMSEA = 0.075 (0.061–0.090). Bollen–Stine *p* = .002 suggested a poor fit. MI’s indicated applying error covariances between item 1 and 2 and item 8 and 9 on the depression factor the model, resulting in a model demonstrating a marginal excellent fit, however, Bollen–Stine *p* = .006, indicated a poor overall model fit. All factor loadings were significant, with the majority having a moderate to high loading onto their respective factor (0.61−0.76), except for item 10, which had a loading of 0.34 on the depression factor. Comparison of both AIC and BIC statistics across the three models revealed that the alternative two-factor, 7 item model demonstrated a superior fit.

Cronbach’s alpha of the depression factor α = 0.80 and for the anxiety factor α = 0.68, and for the total EPDS with 7 items α = 0.82. Coefficient omega was consistent with the alpha values, i.e. ω = 0.80 for the depression factor, ω = 0.69 for the anxiety factor, and ω = 0.81 for the total EPDS. Hierarchical omega coefficient was equally similar with the other reliability measures, i.e. h − ω = 0.81 for the total EPDS and h − ω = 0.79 for the depression sub factor without items 3, 4, and 5.

**Table 2:** Confirmatory factor analyses of the EPDS

Model	*x*^*2*^	*df*	Bollen–Stine *p*	*x*^*2*^*/df*	CFI	TLI	RMSEA [90% CI]	AIC	BIC
1.	66.732**	32	0.005	2.085	0.976	0.967	0.050 [0.033; 0.066]	132.732	134.420
2.	13.257	11	0.234	1.205	0.998	0.995	0.022 [0.00; 0.057]	61.257	62.144
3.	64.990**	32	0.006	2.031	0.978	0.968	0.048 [0.031; 0.065]	130.990	132.679

Model fit indices after applying modifications of error covariances and model comparison information (N = 442)

*CFI* Comparative Fit Index, *TLI* Tucker Lewis Index, *RMSEA* Root Mean Square Error of Approximation, *AIC*Aikaike Information Criterion, *BIC* Bayesian Information Criterion; Model 1 = a 10-item unidimensional model. Model 2 = a 7-item two-factor model. Model 3 = a 10-item two-factor model. **x^2^ significant at *p* < .05

### Research question 2: the utility of the EPDS-3A in detecting anxiety

**Fig. 1:**
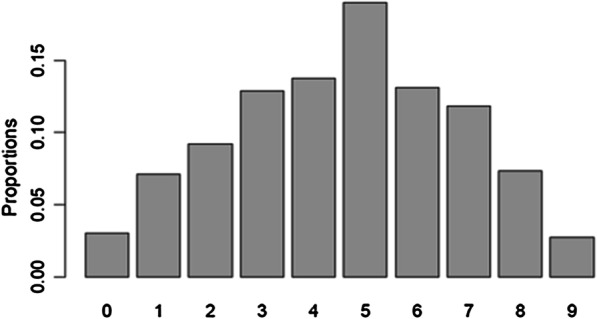
EPDS-3A sores (raw counts)

**Fig. 2:**
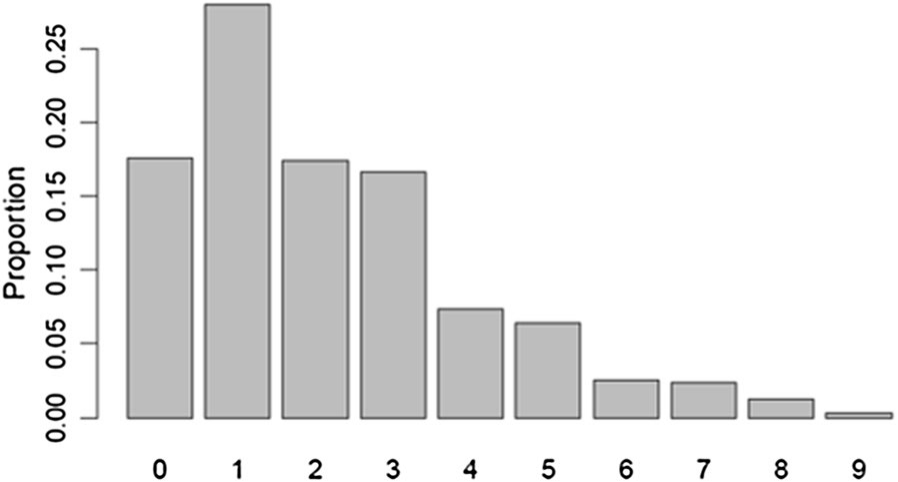
EPDS-3A scores (weighted)

The distribution of raw EPDS-3A scores is presented in Fig. 1. The unusual distribution is due to the sampling strategy. The reweighted distribution, reflecting the distribution in the target population, is presented in Fig. 2.

Table 3 presents sensitivity, specificity, PPV, NPV, and AUC for anxiety caseness status for different EPDS-3A cutoff scores. AUCs was 0.926. Employing our criteria for selecting an optimal cutoff, an EPDS-3A score of ≥ 5 was suggested by the data.

**Table 3:** Sensitivity, specificity, PPV, NPV, and AUC for anxiety^a^ of the EPDS-3A

Cut-off^b^	Sens	95% CI	Spec	95% CI	PPV	95% CI	NPV	95% CI	AUC
3	99.3		70.2		14.5		99.9		0.926
4	74.2		85.4		20.5		98.5		
**5**	**70.9**	0.37; 1.05	**92.2**	0.96; 0.88	**31.7**	0.18; 0.46	**98.4**	0.96; 1.01	
6	52.7		97.6		52.4		97.6		
7	41.4		99.4		78.2		97.1		
8	25.2		99.7		82.2		96.3		
9	4.7		1		86.7		95.4		

* Sens* sensitivity, *95% CI* confidence interval, *Spec* specificity, *PPV* positive predictive value, *NPV* negative predictive value, *AUC* the area under the ROC curve

Values in **bold** represent the most adequate combination of sensitivity, specificity, and PPV. For the sake of clarity 95% CIs provided only for the optimal cutoff-scores. 95% CIs for all values can be provided by request.

^a^Anxiety caseness status defined by a score within the clinical range on the anxiety scale of the Hopkins Symptom Check-List − 63, for which national norms exist [25, 26]

^b^A value score exactly equal to the cut-off is understood as being a case

Table 4 shows the proportion of anxiety cases identified by the EPDS-3A and the total EPDS respectively. The EPDS-3A and the total EPDS work in similar ways with each scale identifying 70.9% of the true anxiety cases. Of the anxiety cases identified by the EPDS-3A, 96.5% were also identified by the total EPDS. Moreover, the EPDS-3A identified 92.2% of true non-cases; this number was 92.7% for the total EPDS. Adding the EPDS-3A to the total EPDS leads to correct identification of an additional 2.5% of the true anxiety cases.

**Table 4:** Comparison of the total EPDS and the EPDS-3A with respect to percentage of identified cases with clinical levels of anxiety

Anxiety caseness status^﻿a^						
	Not case			Case		
	EPDS-3A			EPDS-3A		
	Below cutoff	Above cutoff	Total	Below cutoff	Above cutoff	Total Total EPDS
Total EPDS Below cutoff^﻿b^	90.9%	1.8%	92.7%	26.5%	**2.5%**	29.0%
Total EPDS Above cutoff	1.3%	6.0%	7.3%	2.5%	68.4%	70.9%
Total EPDS-3A	92.2%	7.8	–	29.0%	70.9%	–

All values are calculated on the re-weighted data such that it represents the actual distribution in the target population. The value in **bold** represents the proportion of anxiety cases identified with the EPDS-3A that are not identified by the total EPDS

^a^Anxiety caseness status defined by a score within the clinical range on the anxiety scale of the Hopkins Symptom Check-List − 63, for which national norms exist [25, 26]

^b^A cutoff of 5 or more is used as suggested by the data, see Table 2

## Discussion

The objective of this study was to examine whether the EPDS items 3, 4, and 5 (EPDS-3A) can be used as a time and resource-effective way of screening for anxiety in the postnatal period in settings where the EPDS is already implemented and where implementation of additional instruments to detect anxiety is not feasible.

First, CFAs of the three models indicated that the EFA-driven two-factor structure of the EPDS consisting of a an anxiety factor (items 3, 4 and 5) and a depression factor (items 1, 2, 8 and 9) [15], was a better measurement model for the present data in comparison with a unidimensional model including all 10 original EPDS items and an alternative two-factor model found in a previous study [28] including all 10-items consisting of an anxiety factor (items 3, 4, and 5) and a depression factor including all remaining items. Overall, the different estimates of reliability indicated acceptable internal consistency (indicated by Cronbach’s alpha and construct reliability by coefficient Omega) although estimates for the anxiety factor were just below the generally agreed upon thresholds of 0.70 (e.g., [32, 42].

Taken together, our results confirmed the presence of a sub-factor consisting of items 3, 4, and 5, also found in previous studies [14], suggesting that the EPDS-3A is a stabil phenomena across samples and cultures. Second, in this Danish postnatal sample, the EPDS-3A evidenced an AUC of 0.926 indicating that the EPDS-3A has high discriminative power for detecting clinical levels of anxiety.

A score ≥ 5 was suggested by the data as the optimum cutoff for detecting clinical levels of anxiety. This cutoff fulfilled our criteria for selecting the cutoff with a sensitivity ≥ 70%, specificity ≥ 90%. In comparison, Matthey [16] suggested a cutoff of ≥ 6 to be the appropriate cutoff (sensitivity: 66.7%; specificity: 88.2%, and Swalm et al. ([17] suggested a cutoff of ≥ 4 to be optimal. These differences may be explained by several factors. Swalm et al. [17] did not use an external criteria for validating their cutoff and instead suggested their cutoff because it identified the top-quartile of their sample. In regard to the study by Matthey [16]differences in optimal cutoffs may be explained by differences in sample size and/or the classification of anxiety. In the sample used by Matthey [16], 18 (7.6%) of the mothers presented with anxiety whereas in our sample 161 (30.26%) mothers had clinical levels of anxiety. It should be noted that the high proportion of anxiety cases in our study is due to the oversampling strategy employed and does not represent prevalence rates in the target population. Still, this strategy provided us a with a solid basis for estimating accurate ROCs of the EPDS-3A as compared with the sample used in the Matthey [16] study. Moreover, Matthey [16] used a diagnostic interview when establishing caseness status which is often considered a more ‘conservative’ approach compared with self-report measures, and this may also explain the difference in cutoffs. Finally, cultural differences between Matthey (2008)’s Australian sample and our Danish sample may also explain the differences in cutoff scores.

An important aim of this study was to investigate the proportion of women identified by the EPDS-3A beyond those identified with the total EPDS. Some authors [16–18] have argued that adding the EPDS-3A to routine screening with the EPDS leads to a substantial additional identification of women in need for referral and treatment who might otherwise be overlooked. However, our results suggest that the vast majority (> 95%) of mothers with clinical levels of anxiety identified by the EPDS-3A are also identified using the total EPDS. Using the EPDS-3A alongside the total EPDS score would lead to an additional correct identification of 2.5% of the anxiety cases in a Danish population. Furthermore, it would lead to false identification of 2% of anxiety non-cases. Thus, our results show that implementing the EPDS-3A into routine screening with the EPDS only leads to a minor increase in the percentage of women in need of referral. Based on these results, it could therefore be argued that using extra effort on checking the EPDS-3A score during a routine screening with the EPDS should not be recommended. In terms of identifying anxiety, however, the EPDS-3A does not perform worse than the total EPDS. The total EPDS and the EPDS-3A both correctly identify 71% and miss 30% of the true anxiety cases. While not being a perfect anxiety screening instrument, we propose that the EPDS-3A indeed provides a brief, time-efficient tool to screen for anxiety with high discriminative power (AUC = 0.926) for detecting cases of anxiety if no other screening instruments are available or possible to implement.

Finally, our results show that the total EPDS indeed identifies a large group (≥ 70%) of women with clinical levels of anxiety. Considering our results concerning the EPDS-3A this is not surprising, yet the implications are of importance. Rowe et al. (2008) accordingly showed that the total EPDS detects but does not distinguish anxiety disorders from depression in mothers [43]. In their study, the EPDS correctly identified 28 women (44%) as having major depression, either alone or co-morbid with an anxiety disorder. However, 10 mothers (16%) screening positive on the EPDS only had an anxiety disorder and did not fulfill criteria for a diagnosis of depression. Based on these results it could be argued that it is a limitation of the EPDS that it is not specific to depression. On the other hand, since high levels of co-morbidity have been consistently demonstrated in women with psychological difficulties after birth [11, 44], it could also be argued that it is, in fact, a strength of the instrument that it detects a range of difficulties and not just depression. Otherwise, there would a risk of missing a substantial number of new mothers who need referral and treatment—with potential adverse consequences for the infant. Still, it is important for clinicians to keep in mind that the EPDS identifies a heterogeneous group of women and that a positive screening result may reflect anxiety either as the primary condition or comorbid with depression.

It is a limitation of the current study that only women from urban Copenhagen area were included. This may limit generalizability to the whole population. Further, it could be argued that it is a limitation that we used a self-repport measure (the SCL) to establish anxiety caseness status. While diagnostic interviews are usually considered the ‘gold standard’, it can be questioned whether a diagnosis is more valid for the detection of women in need of referral and treatment than the SCL used in our study. Diagnostic classification is based on diagnostic symptom criteria and not on comparison of quantitative measures of general distress and symptom severity with population norms. The purpose of the SCL-cutoffs for caseness is to indicate the demarcation between normal distress and clinical cases (e.g. clinical anxiety) which in our perspective is in accordance with the purpose of screening for mental health issues in the perinatal women. Moreover, the SCL-63 used in our study is thoroughly validated [25, 26], and the cutoff is based on population norms for adult Danish women.

We also believe that our study has some strengths. Using of an over-sampling strategy ensured high numbers of women scoring within the high range of the EPDS (and in turn the EPDS-3A). Further, the use of sample weighting according to the population wide distribution of the EPDS scores in Danish postnatal women enabled us to re-weigh the sample corresponding to if we had done a random sample of the full population but with substantial higher statistical power.

## Conclusions

In this study of postnatal women, a cutoff of ≥ 5 on the EPDS-3A was found to be efficient for identifying women experiencing clinical levels of anxiety (sensitivity: 70.9%; specificity: 92.2). In settings where the EPDS is already implemented and where adding extra mental health screening instruments is not feasible, the EPDS-3A could be used as an resource-effective means of detecting mothers with possible anxiety disorder. At the same time, adding the EPDS-3A to routine screening with the EPDS only leads to a minor increase in the percentage of women in need of referral because the vast majority of women screening positive on the EPDS-3A also screen positive on the total EPDS. Using the EPDS-3A score along with the total EPDS score can indicate whether a mother may be suffering from anxiety either co-morbid with depression or as the primary problem.

## Supplementary Information

**Additional file 1.** Edinburgh Postnatal Depression Scale.

## Data Availability

The datasets used in the current study are available from the corresponding author on reasonable request.

## Abbreviations

ROCs: receiver operating characteristics
AUC: area under the ROC curve
EFA: exploratory factor analysis
CFA: confirmatory factor analysis
EPDS: The Edinburgh Postnatal Depression Scale
EPDS-3A: Subscale of the Edinburgh Postnatal Depression Scale consisting of items 3,4, and 5
NPV: negative predictive value
PPV: positive predictive value
SCL: Hopkins Symptom Check-List
ISCED: International Standard Classification of Education

## References

[1] Milgrom J, Gemmill AW (2015). Identifying perinatal depression and anxiety: evidence-based practice in screening, psychosocial assessment and management.

[2] O’Connor E, Rossom RC, Henninger M, Groom HC, Burda BU (2016). Primary care screening for and treatment of depression in pregnant and postpartum women: evidence report and systematic review for the US Preventive Services Task Force. JAMA.

[3] Falah-Hassani K, Shiri R, Dennis CL (2017). The prevalence of antenatal and postnatal co-morbid anxiety and depression: a meta-analysis. Psychol Med.

[4] Matthey S, Souter K, Valenti B, Ross-Hamid C (2019). Validation of the MGMQ in screening for emotional difficulties in women during pregnancy. J Affect Disord.

[5] Farr SL, Dietz PM, O'Hara MW, Burley K, Ko JY (2014). Postpartum anxiety and comorbid depression in a population-based sample of women. J Womens Health.

[6] Heron J, O’Connor TG, Evans J, Golding J, Glover V, Team AS (2004). The course of anxiety and depression through pregnancy and the postpartum in a community sample. J Affect Disord.

[7] Buss C, Davis EP, Muftuler LT, Head K, Sandman CA (2010). High pregnancy anxiety during mid-gestation is associated with decreased gray matter density in 6–9-year-old children. Psychoneuroendocrinology.

[8] Kingston D, Tough S, Whitfield H (2012). Prenatal and postpartum maternal psychological distress and infant development: a systematic review. Child Psychiatry Hum Dev.

[9] Polte C, Junge C, von Soest T, Seidler A, Eberhard-Gran M, Garthus-Niegel S (2019). Impact of maternal perinatal anxiety on social-emotional development of 2-year-olds, a prospective study of Norwegian mothers and their offspring. Matern Child Health J.

[10] Falah-Hassani K, Shiri R, Dennis C-L (2016). Prevalence and risk factors for comorbid postpartum depressive symptomatology and anxiety. J Affect Disord.

[11] Meades R, Ayers S (2011). Anxiety measures validated in perinatal populations: a systematic review. J Affect Disord.

[12] Cox JL, Holden JM, Sagovsky R (1987). Detection of postnatal depression. Development of the 10-item Edinburgh Postnatal Depression Scale. Br J Psychiatry.

[13] Hewitt CE, Gilbody SM, Mann R, Brealey S (2010). Instruments to identify post-natal depression: which methods have been the most extensively validated, in what setting and in which language?. Int J Psychiatry Clin Pract.

[14] Kozinszky Z, Töreki A, Hompoth EA, Dudas RB, Németh G (2017). A more rational, theory-driven approach to analysing the factor structure of the Edinburgh Postnatal Depression Scale. Psychiatry Res.

[15] Smith-Nielsen J, Matthey S, Lange T, Væver MS (2018). Validation of the Edinburgh Postnatal Depression Scale against both DSM-5 and ICD-10 diagnostic criteria for depression. BMC Psychiatry.

[16] Matthey S (2008). Using the Edinburgh Postnatal Depression Scale to screen for anxiety disorders. Depress Anxiety.

[17] Swalm D, Brooks J, Doherty D, Nathan E, Jacques A (2010). Using the Edinburgh postnatal depression scale to screen for perinatal anxiety. Arch Womens Ment Health.

[18] Stasik-O’Brien SM, McCabe-Beane JE, Segre LS (2019). Using the EPDS to Identify Anxiety in Mothers of Infants on the Neonatal Intensive Care Unit. Clin Nurs Res.

[19] Fairbrother N, Corbyn B, Thordarson DS, Ma A, Surm D (2019). Screening for perinatal anxiety disorders: room to grow. J Affect Disord.

[20] van der Zee-van den Berg AI, Boere-Boonekamp MM, Groothuis-Oudshoorn CGM, Reijneveld SA (2019). The Edinburgh Postpartum Depression Scale: stable structure but subscale of limited value to detect anxiety. PLoS ONE.

[21] Marteau TM, Bekker H (1992). The development of a six-item short-form of the state scale of the Spielberger State—Trait Anxiety Inventory (STAI). Br J Clin Psychol.

[22] Væver MS, Smith-Nielsen J, Lange T (2016). Copenhagen infant mental health project: study protocol for a randomized controlled trial comparing circle of security–parenting and care as usual as interventions targeting infant mental health risks. BMC Psychol.

[23] Edmondson OJ, Psychogiou L, Vlachos H, Netsi E, Ramchandani PG (2010). Depression in fathers in the postnatal period: assessment of the Edinburgh Postnatal Depression Scale as a screening measure. J Affect Disord.

[24] Levis B, Negeri Z, Sun Y, Benedetti A, Thombs BD. Accuracy of the Edinburgh Postnatal Depression Scale (EPDS) for screening to detect major depression among pregnant and postpartum women: systematic review and meta-analysis of individual participant data. BMJ 2020;371:m4022. 10.1136/bmj.m4022 PMC7656313 33177069

[25] Olsen LR, Mortensen EL, Bech P (2004). The SCL-90 and SCL-90R versions validated by item response models in a Danish community sample. Acta Psychiatr Scand.

[26] Olsen LR, Mortensen E, Bech P (2006). Mental distress in the Danish general population. Acta Psychiatr Scand.

[27] Costello AB, Osborne J (2005). Best practices in exploratory factor analysis: four recommendations for getting the most from your analysis. Pract Assess Res Eval.

[28] Byrne BM (2010). Structural equation modeling with AMOS: basic concepts, applications, and programming (multivariate applications series). N Y Taylor Francis Group.

[29] Nevitt J, Hancock GR (2001). Performance of bootstrapping approaches to model test statistics and parameter standard error estimation in structural equation modeling. Struct Equ Model.

[30] Aguinis H, Gottfredson RK, Joo H (2013). Best-practice recommendations for defining, identifying, and handling outliers. Organ Res Methods.

[31] Yuan K-H, Zhong X (2013). Robustness of fit indices to outliers and leverage observations in structural equation modeling. Psychol Methods.

[32] Phillips J, Charles M, Sharpe L, Matthey S (2009). Validation of the subscales of the Edinburgh Postnatal Depression Scale in a sample of women with unsettled infants. J Affect Disord.

[33] Brown TA (2015). Confirmatory factor analysis for applied research.

[34] Shek DT, Yu L (2014). Use of structural equation modeling in human development research. Int J Disabil Hum Dev.

[35] Dagnall N, Denovan A, Parker A, Drinkwater K, Walsh RS (2018). Confirmatory factor analysis of the inventory of personality organization-reality testing subscale. Front Psychol.

[36] Kyriazos TA (2018). Applied psychometrics: the 3-faced construct validation method, a routine for evaluating a factor structure. Psychology.

[37] Widhiarso W, Ravand H (2014). Estimating reliability coefficient for multidimensional measures: a pedagogical illustration. Rev Psychol.

[38] Watkins MW (2017). The reliability of multidimensional neuropsychological measures: from alpha to omega. Clin Neuropsychol.

[39] Hayes AF, Coutts JJ (2020). Use omega rather than Cronbach’s alpha for estimating reliability. But…. Commun Methods Meas.

[40] Leeflang MMG, Moons KGM, Reitsma JB, Zwinderman AH (2008). Bias in sensitivity and specificity caused by data-driven selection of optimal cutoff values: mechanisms, magnitude, and solutions. Clin Chem.

[41] Bollen KA, Stine RA (1992). Bootstrapping goodness-of-fit measures in structural equation models. Sociol Methods Res.

[42] Tavakol M, Dennick R (2011). Making sense of Cronbach's alpha. Int J Med Educ.

[43] Rowe HJ, Fisher JR, Loh WM. The edinburgh postnatal depression scale detects but does not distinguish anxiety disorders from depression in mothers of infants. Arch Women's Mental Health 2008;11(2):103-8. 10.1007/s00737-008-0003-z. 10.1007/s00737-008-0003-z 18463939

[44] Goodman JH, Tyer-Viola L (2010). Detection, treatment, and referral of perinatal depression and anxiety by obstetrical providers. J Womens Health.

