# Supplementary material for: Can the Edinburgh Postnatal Depression Scale-3A be used to screen for anxiety?
Source: BMC Psychol. 2021 Aug 7;9:118. doi: 10.1186/s40359-021-00623-5 (PMC8349495; doi:10.1186/s40359-021-00623-5)
Supplement: Supplementary file 1 — Additional file 1. Edinburgh Postnatal Depression Scale. [file 40359_2021_623_MOESM1_ESM.pdf]

Edinburgh Postnatal Depression Scale[1] (EPDS)

Name: ______________________________ Address: ___________________________

Your Date of Birth: ____________________ ___________________________

Baby's Date of Birth: ___________________ Phone: _________________________

As you are pregnant or have recently had a baby, we would like to know how you are feeling. Please check the answer that comes closest to how you have felt **IN THE PAST 7 DAYS**, not just how you feel today.

Here is an example, already completed.

I have felt happy:
- ☐ Yes, all the time
- ☒ Yes, most of the time This would mean: "I have felt happy most of the time" during the past week.
- ☐ No, not very often Please complete the other questions in the same way.
- ☐ No, not at all

In the past 7 days:

1. I have been able to laugh and see the funny side of things
   - ☐ As much as I always could
   - ☐ Not quite so much now
   - ☐ Definitely not so much now
   - ☐ Not at all

2. I have looked forward with enjoyment to things
   - ☐ As much as I ever did
   - ☐ Rather less than I used to
   - ☐ Definitely less than I used to
   - ☐ Hardly at all

*3. I have blamed myself unnecessarily when things went wrong
   - ☐ Yes, most of the time
   - ☐ Yes, some of the time
   - ☐ Not very often
   - ☐ No, never

4. I have been anxious or worried for no good reason
   - ☐ No, not at all
   - ☐ Hardly ever
   - ☐ Yes, sometimes
   - ☐ Yes, very often

*5 I have felt scared or panicky for no very good reason
   - ☐ Yes, quite a lot
   - ☐ Yes, sometimes
   - ☐ No, not much
   - ☐ No, not at all

*6. Things have been getting on top of me
   - ☐ Yes, most of the time I haven't been able to cope at all
   - ☐ Yes, sometimes I haven't been coping as well as usual
   - ☐ No, most of the time I have coped quite well
   - ☐ No, I have been coping as well as ever

*7 I have been so unhappy that I have had difficulty sleeping
   - ☐ Yes, most of the time
   - ☐ Yes, sometimes
   - ☐ Not very often
   - ☐ No, not at all

*8 I have felt sad or miserable
   - ☐ Yes, most of the time
   - ☐ Yes, quite often
   - ☐ Not very often
   - ☐ No, not at all

*9 I have been so unhappy that I have been crying
   - ☐ Yes, most of the time
   - ☐ Yes, quite often
   - ☐ Only occasionally
   - ☐ No, never

*10 The thought of harming myself has occurred to me
   - ☐ Yes, quite often
   - ☐ Sometimes
   - ☐ Hardly ever
   - ☐ Never

Administered/Reviewed by ________________________________ Date ______________________________

[1]Source: Cox, J.L., Holden, J.M., and Sagovsky, R. 1987. Detection of postnatal depression: Development of the 10-item Edinburgh Postnatal Depression Scale. *British Journal of Psychiatry* 150:782-786 .

[2]Source: K. L. Wisner, B. L. Parry, C. M. Piontek, Postpartum Depression N Engl J Med vol. 347, No 3, July 18, 2002, 194-199

Users may reproduce the scale without further permission providing they respect copyright by quoting the names of the authors, the title and the source of the paper in all reproduced copies.

Edinburgh Postnatal Depression Scale[1] (EPDS)

Postpartum depression is the most common complication of childbearing.[2] The 10-question Edinburgh Postnatal Depression Scale (EPDS) is a valuable and efficient way of identifying patients at risk for "perinatal" depression. The EPDS is easy to administer and has proven to be an effective screening tool.

Mothers who score above 13 are likely to be suffering from a depressive illness of varying severity. The EPDS score should not override clinical judgment. A careful clinical assessment should be carried out to confirm the diagnosis. The scale indicates how the mother has felt ***during the previous week***. In doubtful cases it may be useful to repeat the tool after 2 weeks. The scale will not detect mothers with anxiety neuroses, phobias or personality disorders.

Women with postpartum depression need not feel alone. They may find useful information on the web sites of the National Women's Health Information Center <www.4women.gov> and from groups such as Postpartum Support International <www.chss.iup.edu/postpartum> and Depression after Delivery <www.depressionafterdelivery.com>.

SCORING

QUESTIONS 1, 2, & 4 (without an *)
Are scored 0, 1, 2 or 3 with top box scored as 0 and the bottom box scored as 3.

QUESTIONS 3, 5-10 (marked with an *)
Are reverse scored, with the top box scored as a 3 and the bottom box scored as 0.

Maximum score: 30
Possible Depression: 10 or greater
Always look at item 10 (suicidal thoughts)

Users may reproduce the scale without further permission, providing they respect copyright by quoting the names of the authors, the title, and the source of the paper in all reproduced copies.

Instructions for using the Edinburgh Postnatal Depression Scale:

1. The mother is asked to check the response that comes closest to how she has been feeling in the previous 7 days.

2. All the items must be completed.

3. Care should be taken to avoid the possibility of the mother discussing her answers with others. (Answers come from the mother or pregnant woman.)

4. The mother should complete the scale herself, unless she has limited English or has difficulty with reading.

[1]Source: Cox, J.L., Holden, J.M., and Sagovsky, R. 1987. Detection of postnatal depression: Development of the 10-item Edinburgh Postnatal Depression Scale. *British Journal of Psychiatry* 150:782-786.

[2]Source: K. L. Wisner, B. L. Parry, C. M. Piontek, Postpartum Depression N Engl J Med vol. 347, No 3, July 18, 2002, 194-199
